# Native Surface Oxides Featured Liquid Metals for Printable Self-Powered Photoelectrochemical Device

**DOI:** 10.3389/fchem.2019.00356

**Published:** 2019-05-22

**Authors:** Yuqing Wang, Yaqi Li, Jingwei Zhang, Jincheng Zhuang, Long Ren, Yi Du

**Affiliations:** ^1^School of Physics, Beihang University, Beijing, China; ^2^BUAA-UOW Joint Research Centre, Beihang University, Beijing, China; ^3^Institute for Superconducting and Electronic Materials (ISEM), Australian Institute for Innovative Materials (AIIM), University of Wollongong, Wollongong, NSW, Australia

**Keywords:** liquid metal, metal oxide, photoelectrochemical, printing electronics, photodetector

## Abstract

Constructing high-performance photo-electrodes by patterning the photo-active semiconducting components with desirable texture and architecture is one of the most promising approaches to achieve the practical and scale-up application of photo-electric or photoelectrochemical (PEC) devices. However, it is a still big challenge to efficiently and effectively handle nano-structural semiconducting materials into intergraded circuit devices, displaying good electric-contact and stability. Here, a facile manufacture strategy for fabricating native metal-oxides based photo-electrodes by directly printing Ga-based liquid metals is explored. The PEC device, functionalized by the native Ga-oxide functional layer, exhibits self-powered photo-detection behaviors and presents fast photo-electric responsibility in response to the simulated Sunlight illumination. This printable PEC device shows good potential for high sensitive self-powered photo-detector and provides a flexible and versatile approach for the design and fabrication of novel electrode structures.

## Introduction

Photoelectrochemical (PEC) devices offer a promising method of converting light into electric power or chemical fuels (Fujishima and Honda, 1972; Li et al., 2013b). Being distinguished from the solid-state junction in the classic photovoltaics devices, multi-junction by contacting the semiconductor with an electrolyte (liquid, gel or organic solid) constructs the internal circuit of the PEC devices (Grätzel, 2001). This type of design enables a facile transfer of photo-induced charge carriers across the semiconductor-electrolyte interface, realizing the migration of electrons from the internal circuit to the external circuit (Hagfeldt and Grätzel, 2000). Specifically, the ionic conduction in the semiconductor-electrolyte system offers an efficient way to develop nanomaterials as photo-responsive media, which largely enhance the specific interface area and carries migrating channels, departing completely from the requirement of perfect solid-solid electrical contact in photovoltaics devices (Sivula and van de Krol, 2016). Due to the large specific surface area, high light-harvest ability and the appearance of quantum confinement effects, semiconducting nanomaterials shows huge potential for building advanced PEC devices, however, effective construction of stable photo-electrodes with these tiny objects are full of challenge (Walter et al., 2010; Lee et al., 2014; Chen et al., 2015).

For general fabrication of photo-electrodes, slurries of photo-active nanomaterials (normally semiconductors) mixed with binders (e.g., Nafion, polyvinylidene fluoride) are spread over the surface of a conductive substrate, which acts as the current collection from internal circuit to external circuit (Govindaraju et al., 2017). Although the addition of polymer binders favors the immobilization of functional nanomaterials on the surface of the current collection, it takes risks that may increase the series resistance, block active sites and inhibit diffusion, leading to reduced utilization and conversion of photo-energy (Qiu et al., 2016; Jia et al., 2019). It is expected that such issues could be solved by direct growth of the photo-active materials on conductive substrates via physical/chemical deposition, or wet chemical synthesis process (Sheng et al., 2019). However, the requirement of experimental conditions including high temperature and excessive chemical treatments, impose serious limitations on the scale-up practical applications.

In the present work, a novel approach is proposed that allows for direct printing high sensitive photo-electrodes by using Gallium-based liquid metal (Ga-based LM) as current collection and its native oxide layer as two-dimensional (2D) photo-active component. The basic idea is that a thin layer of oxide, Ga_2_O_3_, which is an important wide-band-gap semiconductor for photo-electric device (Hou et al., 2006; Yan et al., 2010; Zhang et al., 2015; Qian et al., 2017), forms nearly instantaneously when the galinstan (a eutectic Ga-based LM alloy used in this work) exposure to oxygen at room temperature (Regan et al., 1997; Syed et al., 2017; Zavabeti et al., 2017). This 2D semiconducting layer continually covers on and naturally contacts with the high-electrical-conductive LM matrix, establishing stable and perfect matching in the semiconductor-liquid metal junctions for good electron migration (Sivan et al., 2013; Daeneke et al., 2018). In addition, as metals that are liquid at room temperature, Ga-based LMs enable rapid and facile room temperature processing and can be injected, printed, or even 3D printed on either soft or hard substrates (Ladd et al., 2013; Joshipura et al., 2015), forming highly conductive, durable, and stretchable components in soft electronics (Dickey, 2017; Wang et al., 2018). With these advantages, 2D semiconducting Ga_2_O_3_ has been successfully automatically patterned on a metallic soft substrate with desirable texture and architecture while printing the Ga-based liquid metal into electronic circuits, which is expected to controllably regulate the light-harvest and achieve high efficient light-electric conversion. The as-prepared flexible photo-electrodes, after enveloping in a solid-state electrolyte-based PEC cell, exhibit fast photo-electric responsibility in response to the simulated Sunlight illumination, even at a very irradiation density (20 mW/cm^2^), presenting a good potential for printable high-sensitive photo-detectors. The proposed strategy and the constructed PEC devices pave the way to the scale-up manufacture and application of PEC devices, which also offer a promising extension for other electrochemical devices.

## Experimental Section

### Fabrication of Ga-Based LM and Related Circuits

The Ga-based LM used in this work is galinstan bulk sample, which is prepared by the co-melting methods with the weight ratio: 68% Ga, 22% In, and 10% Sn. Printing galinstan into desired patterns was fabricated using a layered molding and casting process. The substrate is a silicone rubber made by spin coating the polydimethylsiloxane (PDMS) mixture onto glass slides.

### Characterizations of Structure and Properties

Optical microscope observations were performed using Leica DM6000 Optical microscope. Field emission scanning electron microscope (FE-SEM) observations were performed using a JEOL JSM-7500FA microscope with an EDX solid-state X-ray detector. Transmission electron microscope (TEM) images, selected area electron diffraction (SAED) patterns were obtained using a JEOL JEM-2011. The surface roughness of the sample together with the thickness was analyzed using a JPK Nanowizard atomic force microscope (AFM) in the tapping mode. The crystal structure of printed galinstan pattern was evaluated by X-ray diffraction (XRD) (GBC MMA diffractometer) using Cu Kα radiation. The UV-vis absorption spectra were measured by the means of the diffuse reflection mode using a Shimadzu 2550 UV-vis spectrometer equipped with an integrating sphere.

### Fabrication of PEC Device

The PEC device here is mainly composed by the printed galinstan circuit on PDMS as working electrode, the Pt deposited on the surface of PDMS as contrast electrode, and the neutral solid-state electrolyte. The neutral solid electrolyte was prepared by a water bath method. In detail, 1.2 g of sodium chloride (NaCl) and 1 g of polyvinyl alcohol (PVA) were added to 20 mL of deionized water. The mixture was kept stirring at 90 °C in a water bath environment for about 30 min until a clear colloidal gel was obtained. Then, the colloidal electrolytes were naturally cooled and sit overnight until the bubbles were completely eliminated. After depositing Pt with desired sharp with 1 cm distance to the galinstan pattern on the PDMS substrate, a droplet of NaCl-PVA gel was spread over the surface of PMDS connecting the working electrode and contrast electrode. In the final step, a transparent thin layer of PDMS was spread the whole device to separate the whole device to the outside atmospheric environment. Only two conductive wire connected inside electrodes with the outside, providing the signal output.

### Photo-Response Activities Measurements

All photoresponse performance tests are based on a standard two-electrode electrochemical workstation (CHI660D, CH Instruments, Inc., Shanghai). During the test, the electrode clips of the counter electrode and the reference electrode were clamped at the same conductive wire connecting Pt, and the working electrode was clamped at the other wire connecting printed galinstan pattern. The linear sweep voltammogram (LSV) was measured at a scan rate of 10 mV·s^−1^. The current–time relationship (i–t) curve was tested by periodically turning on the “on” and “off” light source states at a frequency of 10 s. A 350 W xenon arc lamp was placed at a distance of 20 cm from the device as a light source for testing. The light only irradiated on the surface of galinstan electrode, and illumination intensity at the location of the photo-anode was measured and tuned before the test. All the photo-response current density has been normalized to the dark current density, in order to clearly compare the variation with and without light irradiation.

## Results and Discussions

**Figure 1:**
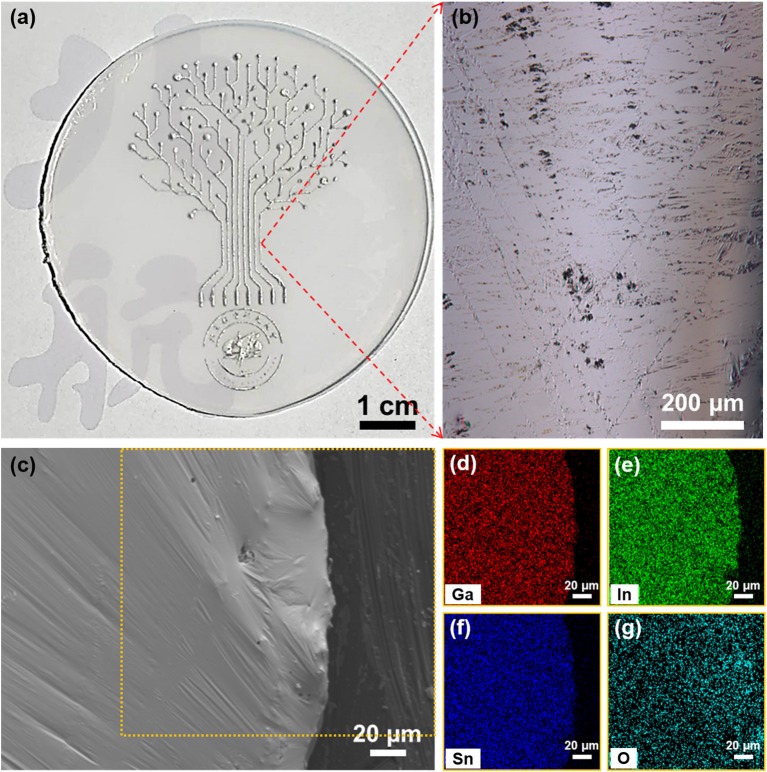
**(a)** Digital image of a printed galinstan pattern. **(b)** Optical microscope image of a local area marked in **(a)**. **(c)** SEM of the edge area of the printed galinstan pattern in **(a)**, and **(d)–(g)** the corresponding EDX mapping results in **(c)**; (**(d)** for Ga, **(e)** for In, **(f)** for Sn, and **(g)** for O, respectively).

By using the galinstan for printing electronics, various defined patterns can be fabricated on a substrate, as demonstrated in Figure 1a. The as-printed circuits presented typical metallic color and show high electric conductivity. As expected, a smooth surface, featured by reflecting the visible light like a mirror was observed from the optical microscope (Figure 1b). Meanwhile, small thin flakes which may be the native oxide layer have been also found on the surface of galinstan. According to the further SEM characterization for the edge area of the printed galinstan circuit, one can find that smooth surface with several ripples displaying in Figure 1c, which is similar with a flexible and continues “skin” covering a soft flat core. The EDX mapping (Figures 1d–g) presents a homogenous distribution of Ga, In, and Sn in the selective square area in Figure 1c, and the mass proportion of Ga, In, and Sn is the same with the weight proportions in galinstan bulk used for printing. The signal for O was also detected everywhere, indicating the homogenous distribution of O in the surface of galinstan pattern, and substrate. These microscope characterizations indicate that a thin oxide layer grows on the whole surface of galinstan LM, and those small pieces of sheet shown in optical microscope image are regarded as the thicken oxide film that cracks from the area undergoing mechanical agitation during the printing process.

**Figure 2:**
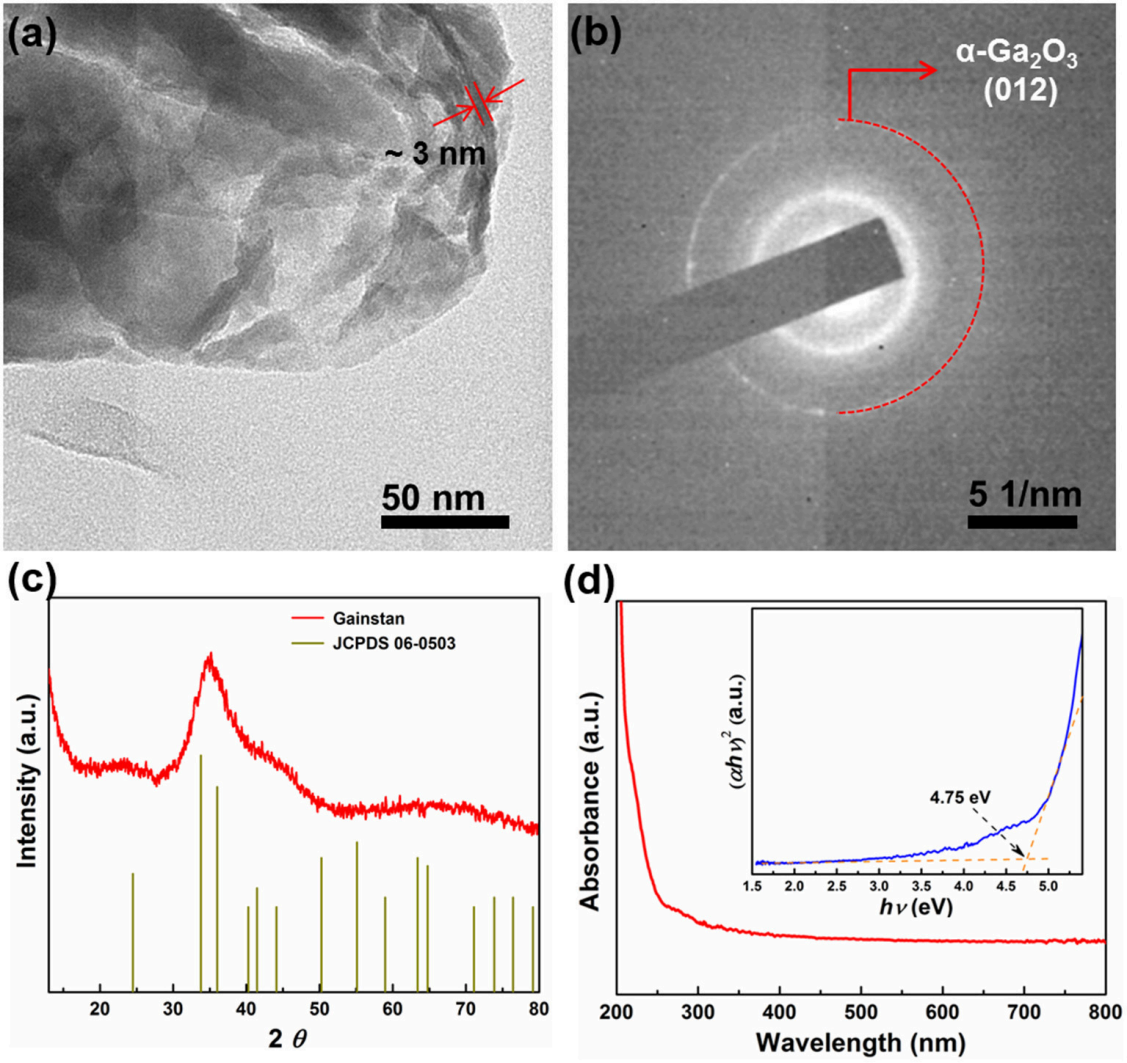
**(a)** TEM image and **(b)** SAED pattern of the native Ga-oxide nanosheets. **(c)** XRD pattern of the galinstan electrode. **(d)** The absorption spectrum of the native Ga-oxide nanosheets, the inset is (α*h*ν)^2^ vs. *h*ν.

To reveal the chemical composition of the native oxide layer, typical XRD was conducted and the patterns of the printed galinstan LM are shown in Figure 2c. Although there is no obvious sharp peak observed indicating poor crystallization of the sample, all of the observed broad diffraction peaks are in good agreement with the reported diffraction peaks of α-Ga_2_O_3_ (JCPDS no. 06-0503), a hexagonal system with lattice constants a = b = 4.97 Å, c = 13.42 Å. Further characterization on the morphology and structure of this 2D Ga-oxide layer has been investigated by electron diffraction and high-resolution TEM (HRTEM). Taking the exfoliation method (Daeneke et al., 2017; Zavabeti et al., 2017) to deposit the Ga-oxide nanosheets onto the TEM grids, high-magnified TEM images and selected area electron diffraction (SAED) patterns have been recorded. As shown in Figure 2a, the sheet-like morphology featured with many creases or folds illustrates the native Ga-oxide nanosheets on the printed circuit are evidently very thin (~ 3 nm). The thickness of the native Ga-oxide nanosheets was further measured by AFM (Figure S1) by transferring the nanosheets on a SiO_2_/Si substrate. The stacks of several nanosheets with smooth surface was observed, a thickness of about 3.6 nm was estimated from the line profile depicted in an individual sheet. The SAED pattern, displayed in Figure 2b, presents a clear ring which can be ascribed to the (012) planes of the hexagonal α-Ga_2_O_3_ structure, indicates that the Ga-oxide nanosheet crystallizes in small domains that may be not aligned with each other, similar to the reported oxidation behavior of other liquid metal in oxygen-rich environments (Daeneke et al., 2017).

As the photo-active component of the constructed PEC device, the optical absorption properties of the native Ga-oxide nanosheets samples were explored by UV-vis absorption spectroscopy, displayed in Figure 2d. It shows that the absorption onset of the Ga-oxide nanosheets samples is at around 260 nm, exhibiting strong absorption in the UV region. The corresponding optical bandgap can be estimated by Tauc's analysis based on the equation (α*h*ν)^2^ = A(*h*ν – *E*_g_), to be ~ 4.75 eV, which is consistent with the previous reports (Li et al., 2013a; Zhang et al., 2015; Syed et al., 2017).

**Figure 3:**
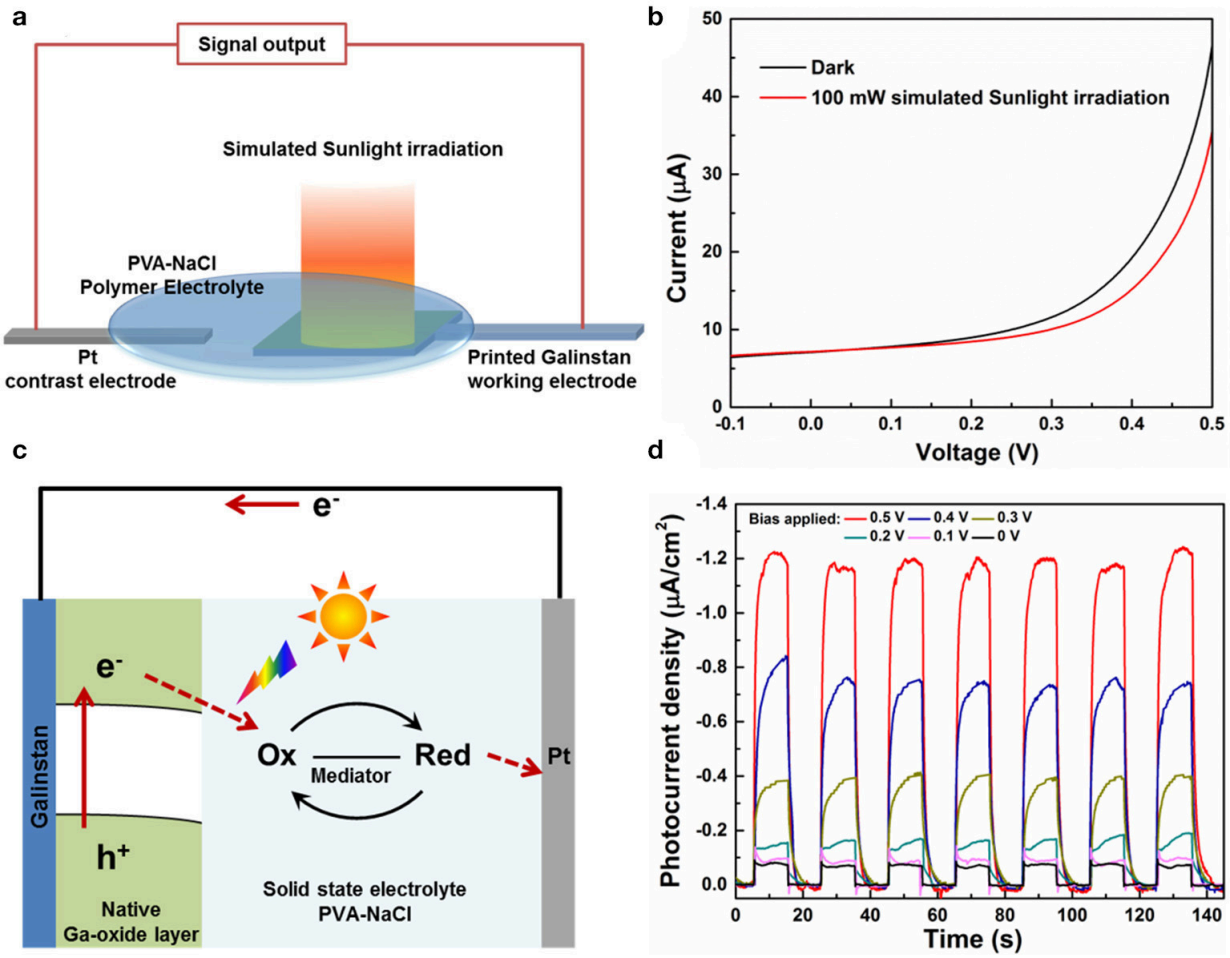
**(a)** Schematic illustration of the PEC device and test system. **(b)** Current-voltage relationship (I–V) of the printed galinstan electrode. **(c)** Schematic illustration of the working mechanism of the as-constructed PEC device. **(d)** Normalized photocurrent density of printed galinstan PEC device at different bias potentials (0 V, 0.1 V, 0.2 V, 0.3 V, 0.4 V, 0.5 V, vs. the Pt electrode) with the on-off switching operation of 100 mW·cm^−2^ simulated Sunlight irradiation.

The photo-response performance of the printed galinstan photo-electrode was evaluated by PEC tests, the schematic illustration of the PEC device and test system are shown in Figure 3a. The linear sweep voltammetry (LSV) curves of as-printed galinstan photo-electrode were required at a scanning speed of 10 mV·s^−1^, in both light and dark conditions. As shown in Figure 3b, it is obvious that the anode current of Ga-oxide layer under simulated Sunlight illumination is lower than the dark current, and the current intensity of the anode decreased with bias potential in contrast to the Pt electrode increasing from 0 to 0.5 V, which matched the character of a p-type semiconductor (Wang et al., 2017). Thus, it reveals that this native Ga-oxide layer is a p-type semiconductor, which is also identical to previous report (Shafiei et al., 2017). Photocurrent is defined as the photo-induced change in the currents with and without light irradiation. Therefore, for the PEC device constructed in this work, the photocurrent in response to the light is actually the decreasing value of the anode current in dark condition and light irradiation condition.

Using this definition of the photocurrent, as shown in Figure 3d, we tested the photocurrent density of the printed galinstan electrode under different bias potentials from 0 to 0.5 V in contrast to the Pt electrode. The photocurrent of the sample increased in negative direction rapidly and saturated in a short time under irradiation, and this photo-responsive switching behavior can be steadily reproduced via periodically turning the “on” and “off” state with a 10 s frequency. It was found that the negative photocurrent density at high bias potentials is much enhanced in comparison to that at low bias potentials, indicating an effective separation of photo-induced electron-hole pairs and a rapid transfer of photo-generated carriers when applying high bias potentials. Interestingly, under the irradiation of light intensity of 100 mW·cm^−2^, the printed galinstan photo-electrode possesses a negative photo-response current of approximately 80 nA·cm^−2^ and excellent photo-response rate (< 0.1 s) without applying bias voltage (0 V). This indicates that the constructed PEC device exhibits self-powered photo-detection behavior without relying on external power supplies. Figure 3c illustrates the working mechanism of this PEC device under the irradiation of light. As a p-type semiconductor, the Fermi level at the surface of Ga-oxide is shifted close to the valence band, resulting in a downward band bending (Jiang et al., 2017). Due to the band bending, the photo-generated electrons in p-type Ga-oxide migrate toward the semiconductor-liquid interface to unleash the reduction reaction. Meanwhile, the holes in the valence band of p-type electrode recombine with the outside connection via ohmic contact between both photo-electrodes. In our experiment design, the electrons flow from the working electrode (galinstan) to the counter electrode (Pt) via an external circuit without any bias applied in dark environment, due to the standard reduction potentials of the elements (Ga, In, Sn) in galinstan is lower than Pt (Hoshyargar et al., 2017). Therefore, under the irradiation of light, the photo-induced migration of carriers would reduce the current density in the external circuit, resulting in the decreased current intensity under simulated Sunlight illumination shown in Figure 3b.

**Figure 4:**
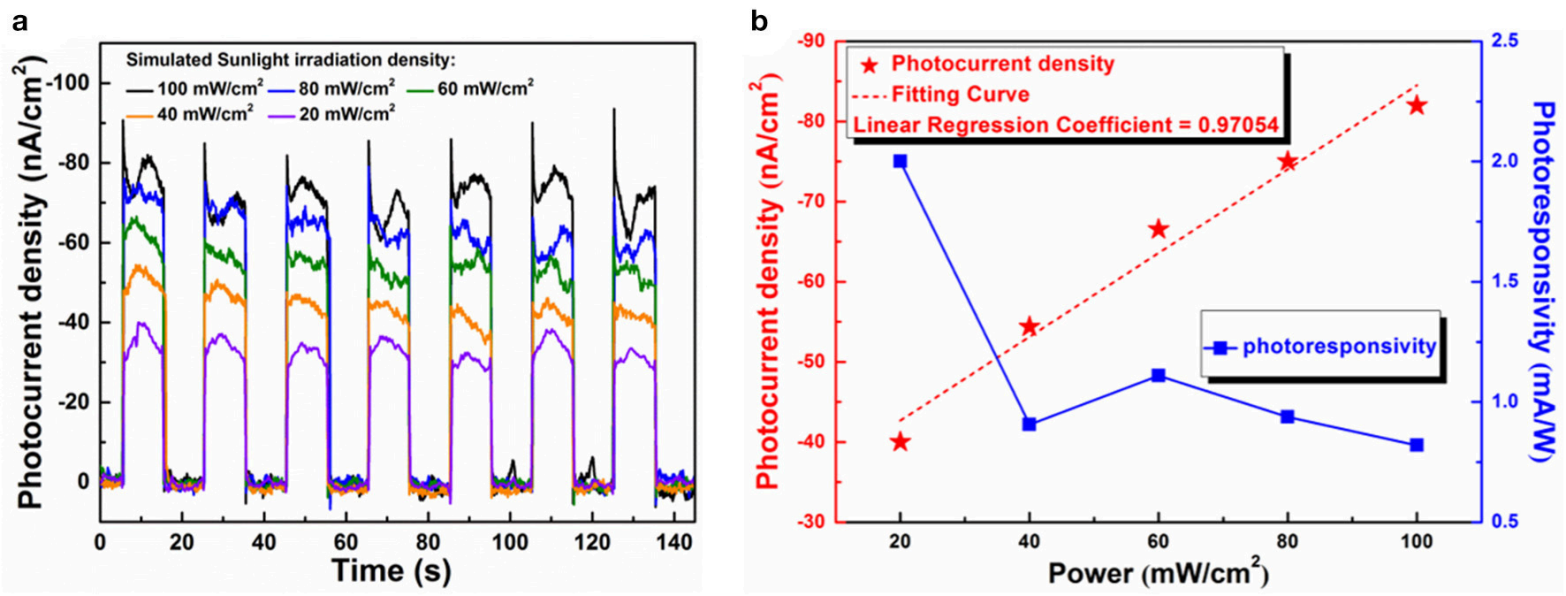
**(a)** Photocurrent density of printed galinstan PEC device under different power intensity at 0 V bias potential (20 mW·cm^−2^, 40 mW·cm^−2^, 60 mW·cm^−2^, 80 mW·cm^−2^, 100 mW·cm^−2^). **(b)** Photocurrent density and calculated responsivity as a function of the power intensity at 0 V bias potential.

The influence of light intensity on the photocurrent density is an important factor in evaluating the photo-response ability of a photo-electrode, which can reflect the sensitivity of the photo-detector constructed by PEC devices. Figure 4a shows the photo-response switching behaviors of the as-prepared PEC device under different illumination intensities without applied voltage. The photo-response rate remained high (< 0.1 s) even for the low light irradiation density of 20 mW·cm^−2^, and the photocurrent of the device increases in a negative direction with the increase of the light densities, exhibiting the self-powered characteristic again. As shown in Figure 4b, the negative photocurrent density appears linearly increases with a rise in illumination intensity over a wide range from 20 to 100 mW·cm^−2^. In order to further evaluate the photo-response performance of the as-printed PEC photodetector, the values of responsivity of the 2D native Ga-oxide nanosheets based photodetector can be obtained through the following equation (Qiao et al., 2019): R = I/J _light_, where the I is the photocurrent density (nA·cm^−2^) and J _light_ is the illumination intensity (mW·cm^−2^). The relationship among the photocurrent density, the photocurrent responsivity and irradiation power intensity is demonstrated in Figure 4b. The photocurrent responsivity of the printed galinstan PEC photodetector varies from 2 to 1 μA/W, showing superior sensitivity in contrast to similar PEC device manufacturing by conventional methods (Chen et al., 2018; Huang et al., 2018).

## Conclusion

In summary, a novel design for printing semiconducting Ga oxides-based PEC devices in desirable texture and architecture was demonstrated. The 2D Ga-oxide semiconductor, as the photo-active materials in this PEC device, can be automatically synthesized by native oxidation of the fresh surface of the galinstan LM during the printing manufacturing process. The natural contact between the semiconducting photo-active material and the conductive soft metal substrate offers good electric communication from the PEC internal circuit to the external. The native Ga-oxide exhibits promising photo-absorbance ability, and the as-printed PEC device exhibits good photo-response performance. Meanwhile, this PEC device as photodetector achieves self-powered photo-detection behaviors and presents high responsibility without external power supplies even for a low light density. This novel design provides strong prospects for the native metal oxide layer of LMs as promising candidates for developing practical printable photodetectors and offers a new platform for manufacturing more complex and soft PEC devices or semiconductor-based photo-electronics.

## Author Contributions

LR and YD designed this project. YW, YL, and JwZ carried out the material preparation and electrochemical test. JcZ carried out and analyzed the XRD, SEM, and TEM results. LR, YD, and YW wrote the paper. All authors discussed the results and revised the manuscript.

### Conflict of Interest Statement

The authors declare that the research was conducted in the absence of any commercial or financial relationships that could be construed as a potential conflict of interest. The handling editor declared a shared affiliation, though no other collaboration, with the authors YD, LR at time of review.
