# Supplementary material for: Native Surface Oxides Featured Liquid Metals for Printable Self-Powered Photoelectrochemical Device
Source: Front Chem. 2019 May 22;7:356. doi: 10.3389/fchem.2019.00356 (PMC6541107; doi:10.3389/fchem.2019.00356)
Supplement: Supplementary file 1 [file Image_1.pdf]

Supplementary Material

Native surface oxides featured liquid metals for printable self-powered photoelectrochemical device

Yuqing Wang[1,2], Yaqi Li[1,2], Jingwei Zhang[1,2], Jincheng Zhuang[1,2], Long Ren[2,3*], Yi Du[1,2,3*]

[1] School of Physics, Beihang University, Beijing 100191, China

[2] BUAA-UOW Joint Research Centre, Beihang University, Beijing 100191, China

[3] Institute for Superconducting and Electronic Materials (ISEM), Australian Institute for Innovative Materials (AIIM), University of Wollongong, Wollongong, NSW 2500, Australia

*** Correspondence:**
Long Ren: lr289@uowmail.edu.au; Yi Du: yi_du@uow.edu.au

1. Supplementary Figures

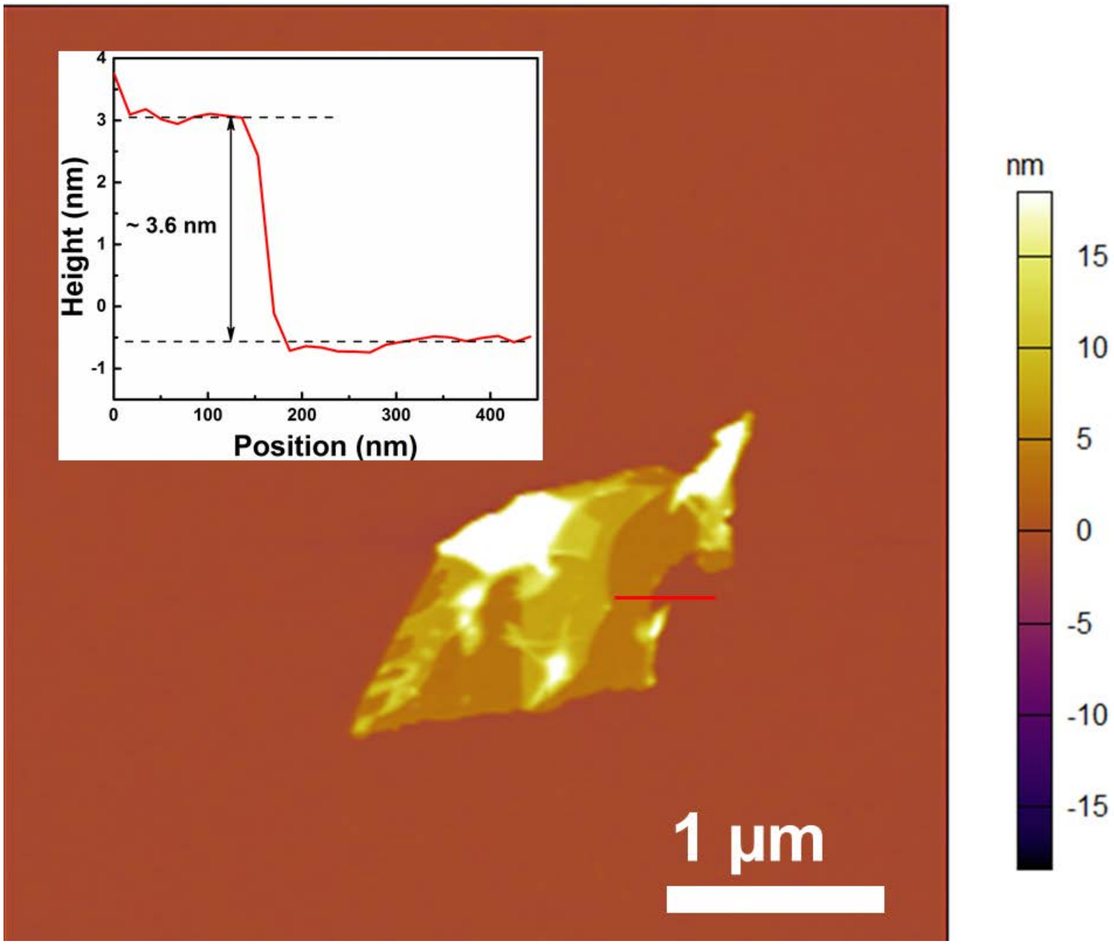

Supplementary Figure S1. AFM images with corresponding thickness profiles (inset) on SiO_2/Si wafer for the small piece of native Ga-oxide from the surface of liquid metal. The linear profile is highlighted by red line depicted in the corresponding position.
